# Supplementary material for: Implementation and evaluation of an electronic consult program at a large academic health system
Source: PLoS One. 2024 Sep 12;19(9):e0310122. doi: 10.1371/journal.pone.0310122 (PMC11392322; doi:10.1371/journal.pone.0310122)
Supplement: S1 Fig — (DOCX) [file pone.0310122.s001.docx]

S1 Figure. Primary Care Provider Survey

PCP Survey

Please complete the following questions about your experience with eConsults.

Q1 Which clinical site(s) do you practice in? Select all that apply.

- Cornell Scott Hill Health Center
- Fair Haven Community Health Center
- Yale Health
- Cornell Scott Hill Health Center
- Fair Haven Community Health Center
- HAVEN Free Clinic
- NEMG
- Oncology
- Yale Health
- Yale Internal Medicine Associates
- Cornell Scott Hill Health Center
- Fair Haven Community Health Center
- NEMG
- Yale Health
- YNH & Fair Haven School-Based Clinic

Q2 Have you ever placed an eConsult?

- Yes
- No

Display This Question:

If Have you ever placed an eConsult? = No

Q3 Please tell us more about why you have not placed any eConsults. Check all that apply.

- I prefer to contact the specialist via other methods (by phone, email or inbasket message in Epic).
- It is easier to place a standard referral visit to the specialist.
- I am not aware of the eConsult option.
- I don't have enough time.
- I forget to use eConsults.
- Other, please explain: ________________________________________________

Display This Question:

If Have you ever placed an eConsult? = No

Q4 What would make you want to use eConsults? Please explain.

________________________________________________________________

Display This Question:

If Have you ever placed an eConsult? = Yes

Q5 Please rate your level of agreement with the following statements.

|  | Strongly Disagree (1) | Disagree (2) | Neither Agree nor Disagree (3) | Agree (4) | Strongly Agree (5) |
| --- | --- | --- | --- | --- | --- |
| I am highly satisfied with the eConsult program. |  |  |  |  |  |
| eConsults are easy to use. |  |  |  |  |  |
| eConsults improve my ability to treat specialty conditions. |  |  |  |  |  |
| eConsults improve overall quality of care. |  |  |  |  |  |
| The guidance in eConsult orders is helpful. |  |  |  |  |  |

Display This Question:

If Have you ever placed an eConsult? = Yes

Q6 How do you most commonly communicate eConsult recommendations to your patients?

- Phone call by nursing team or other support staff
- Phone call by me, the provider who placed the eConsult
- MyChart message
- Other, please specify: ________________________________________________

Display This Question:

If Have you ever placed an eConsult? = Yes

Q7 How much time do you typically spend following up on eConsult recommendations?

- Less than 5 minutes
- 5-10 minutes
- 11-20 minutes
- 21-30 minutes
- More than 30 minutes

Display This Question:

If Have you ever placed an eConsult? = Yes

Q8 Consider the eConsults that you have placed. If eConsults were not an option, what would your alternate first step have been to address questions? Check all that apply.

- Contact specialist via pager/phone
- Contact specialist via inbasket message in Epic
- Contact specialist via email (outside of Epic)
- Search medical reference/clinical guidelines
- Order a standard referral visit to the specialist
- Other ________________________________________________

Display This Question:

If Have you ever placed an eConsult? = Yes

Q9 What are the strengths of eConsults?

________________________________________________________________

Display This Question:

If Have you ever placed an eConsult? = Yes

Q10 What are the weaknesses of eConsults?

________________________________________________________________

Display This Question:

If Have you ever placed an eConsult? = Yes

Q11 If you had a magic wand, what would you change about eConsults?

________________________________________________________________

Display This Question:

If Have you ever placed an eConsult? = Yes

Q12 What do you think threatens the success of eConsults?

________________________________________________________________

Q13 We are also seeking your feedback to improve the referral process in general including communication between primary care and specialties. Thinking about when your patient needs to see a specialist, what barriers have you faced with referrals to specialty care?

________________________________________________________________

Q22 Have you found condition specific guidance within referral orders to be helpful?

- Yes
- No, please explain: ________________________________________________
- I have not noticed this guidance.
