# Supplementary material for: Implementation and evaluation of an electronic consult program at a large academic health system
Source: PLoS One. 2024 Sep 12;19(9):e0310122. doi: 10.1371/journal.pone.0310122 (PMC11392322; doi:10.1371/journal.pone.0310122)
Supplement: S2 Fig — (DOCX) [file pone.0310122.s002.docx]

S2 Figure. Specialist survey

Specialist eConsult Survey

Please complete the following questions about your experience with eConsults.

Q1 What is your specialty?

ADULT

- Allergy
- Cardiology
- Dermatology
- Endocrinology
- Gastroenterology
- Geriatrics
- Hematology
- Hepatology
- Infectious Disease
- Maternal Fetal Medicine
- Nephrology
- Neurology
- Ophthalmology
- Orthopedics
- Otolaryngology
- Psychiatry
- Pulmonary
- Rheumatology
- Sleep Medicine
- Urology

PEDIATRICS

- Allergy
- Cardiology
- Dermatology
- Endocrinology
- Gastroenterology
- Hematology Oncology
- Infectious Disease
- Nephrology
- Neurology
- Orthopedics
- Pulmonary
- Rheumatology
- Urology

Q2 How many years have you been practicing in your specialty (post residency/fellowship)?

________________________________________________________________

Q3 Please estimate the number of eConsults that you have addressed.

- None
- 1-25
- 26-50
- 51-75
- 76-100
- 100+

Q4 Please rate your level of agreement with the following statements.

|  | Strongly Disagree (1) | Disagree (2) | Neither Agree nor Disagree (3) | Agree (4) | Strongly Agree (5) |
| --- | --- | --- | --- | --- | --- |
| I am highly satisfied with the eConsult program. |  |  |  |  |  |
| eConsults improve overall quality of care. |  |  |  |  |  |
| Primary care providers ask appropriate eConsult questions. |  |  |  |  |  |
| I often do not have enough information to answer the question. |  |  |  |  |  |
| Use of eConsults has improved my communication with primary care physicians. |  |  |  |  |  |
| The eConsult program is improving access to in-person appointments in my department. |  |  |  |  |  |
| eConsults are burdensome to address. |  |  |  |  |  |

Q5 What motivates you to answer eConsults? Check all that apply.

- Educate primary care providers
- Reduce unnecessary referrals
- Improve patient care
- Satisfy request from leadership
- Other, please explain: _______________________________________________

Display This Question:

If Please rate your level of agreement with the following statements. = Primary care providers ask appropriate eConsult questions. [ Strongly Disagree ]

Or Please rate your level of agreement with the following statements. = Primary care providers ask appropriate eConsult questions. [ Disagree ]

Q6 Please describe a scenario(s) in which you received an inappropriate eConsult.

________________________________________________________________

Display This Question:

If Please rate your level of agreement with the following statements. = eConsults are burdensome to address. [ Agree ]

Or Please rate your level of agreement with the following statements. = eConsults are burdensome to address. [ Strongly Agree ]

Q7 Please elaborate on why you find eConsults burdensome.

________________________________________________________________

Q8 Please share any comments or suggestions you have about your experience answering eConsults and any ideas for improvement.

________________________________________________________________
