# Supplementary material for: Implementation and evaluation of an electronic consult program at a large academic health system
Source: PLoS One. 2024 Sep 12;19(9):e0310122. doi: 10.1371/journal.pone.0310122 (PMC11392322; doi:10.1371/journal.pone.0310122)
Supplement: Attachment — Submitted filename: Arora response to Reviewers 8-9.docx [file pone.0310122.s003.docx]

**Author Response Letter:**

**Title:** Implementation and evaluation of an electronic consult program at a large academic health system

**REVIEWER COMMENTS:**

Reviewer #1:

1. Is the manuscript technically sound, and do the data support the conclusions?

The manuscript must describe a technically sound piece of scientific research with data that supports the conclusions. Experiments must have been conducted rigorously, with appropriate controls, replication, and sample sizes. The conclusions must be drawn appropriately based on the data presented.

Reviewer #1: Partly

Response: Please see modifications described in the following sections.

2. Has the statistical analysis been performed appropriately and rigorously?

Reviewer #1: No

Response: Thank you for this point. We provided descriptive data (e.g., eConsult volumes, provider satisfaction) that was used to support implementation and evaluation of our electronic consultation (eConsult) program. We hope that our practical approach to describing our experience may help other health systems design, implement and evaluate their eConsult programs.

3. Have the authors made all data underlying the findings in their manuscript fully available?

The [PLOS Data policy](https://nam12.safelinks.protection.outlook.com/?url=http%3A%2F%2Fwww.plosone.org%2Fstatic%2Fpolicies.action%23sharing&data=05%7C02%7Crenee.fekieta%40yale.edu%7C1d52593bc4fc4eb90d9808dcaa7ad586%7Cdd8cbebb21394df8b4114e3e87abeb5c%7C0%7C0%7C638572692034321488%7CUnknown%7CTWFpbGZsb3d8eyJWIjoiMC4wLjAwMDAiLCJQIjoiV2luMzIiLCJBTiI6Ik1haWwiLCJXVCI6Mn0%3D%7C0%7C%7C%7C&sdata=N5TWS99u%2FHkw8oU1hx9zISdxxGJ01yKkeIP8yEsGUVg%3D&reserved=0) requires authors to make all data underlying the findings described in their manuscript fully available without restriction, with rare exception (please refer to the Data Availability Statement in the manuscript PDF file). The data should be provided as part of the manuscript or its supporting information, or deposited to a public repository. For example, in addition to summary statistics, the data points behind means, medians and variance measures should be available. If there are restrictions on publicly sharing data—e.g. participant privacy or use of data from a third party—those must be specified.

Reviewer #1: No

Response: Thank you for bringing this to our attention. We added numerators and denominators to supplement percentages from survey data summarized in Table 2 (PCP and Specialist Satisfaction) and Table 3 (Alternatives to eConsults). No changes were necessary for Table 1 as the data are summarized in raw form to show our institution’s eConsult volumes for context.

4. Is the manuscript presented in an intelligible fashion and written in standard English?

PLOS ONE does not copyedit accepted manuscripts, so the language in submitted articles must be clear, correct, and unambiguous. Any typographical or grammatical errors should be corrected at revision, so please note any specific errors here.

Reviewer #1: Yes

Response: Thank you very much.

5. Review Comments to the Author

- Reviewer #1: The topic is overall interesting; however, some suggestions are necessary:
  The abstract is very short and could be improved to be more structured and more insightful to the reader.

Response: We have added structure and additional detail to the abstract in lines 21-35.

“Background: Although the benefits of eConsults in increasing access and lowering unnecessary utilization have been well described, the development of a successful program can be challenging.

Objective: We sought to share the experiences of a large academic health system in implementing and evaluating a high-volume electronic consultation (eConsult) program across 34 adult and pediatric medical and surgical specialties.

Methods: Using a multi-method approach, we collected qualitative and quantitative data on operational and process outcomes to describe adoption of eConsults, and survey data to capture satisfaction and experience among referring and specialist clinicians.

Results and Conclusions: Data evaluating this eConsult program demonstrated robust uptake of the eConsult workflow as well as high satisfaction amongst primary care and specialty clinicians. Effective implementation strategies included engaging leadership, building a dedicated team, and developing quality assurance mechanisms. These experiences and findings may inform implementation at health systems interested in eConsult programs.”

- Please clarify what you mean by "specialty input"

Response: Thank you for the clarification. This is referring to guidance from a specialist in patient care. We have clarified the language in lines 38-40.

“In this setting, electronic consults (eConsults), asynchronous exchanges between clinicians in the electronic medical record (EMR), have increasingly been used to obtain specialist input in a patient’s care.”

- With regards to the Methods section, most of the information does not belong to this section as it already narratively reports findings. This information can be shortened and reported in the Results section.

Response: In the Methods section under the subheading “Engagement of key stakeholders, collection of early feedback, and adjustment of the model”, we removed this section (after line 141) as we decided these early implementation data did not add value to the manuscript.

- Overall, a key point to be addressed is that the study design of choice (e.g.mixed-methods study?) should be made more clear from the beginning, otherwise the narrative reporting of this experience, however interesting, falls short of an appropriate structure and methodology.

Response: We have clarified this in the abstract: “Using a multi-method approach, we collected qualitative and quantitative data on operational and process outcomes to describe adoption of eConsults, and survey data to capture satisfaction and experience among referring and specialist clinicians.”

- With regards to the Surveys, how were they developed, were they validated? It is important to detail this process in the methods section. Also, the surveys should be made fully available.

Response: The surveys were not validated but rather developed to gather rapid feedback from providers and inform program improvement. We did a careful review of other similar surveys available from the AAMC and other sites using eConsults. As a team, we adapted these other surveys to suit our institution’s needs. In the Methods, under the subheading “Program evaluation – PCP and specialist experiences”, we added a sentence (line 254) to reflect our survey design process. We have also made the surveys available.

6. PLOS authors have the option to publish the peer review history of their article ([what does this mean?](https://nam12.safelinks.protection.outlook.com/?url=https%3A%2F%2Fjournals.plos.org%2Fplosone%2Fs%2Feditorial-and-peer-review-process%23loc-peer-review-history&data=05%7C02%7Crenee.fekieta%40yale.edu%7C1d52593bc4fc4eb90d9808dcaa7ad586%7Cdd8cbebb21394df8b4114e3e87abeb5c%7C0%7C0%7C638572692034327697%7CUnknown%7CTWFpbGZsb3d8eyJWIjoiMC4wLjAwMDAiLCJQIjoiV2luMzIiLCJBTiI6Ik1haWwiLCJXVCI6Mn0%3D%7C0%7C%7C%7C&sdata=F%2BEIMAb77ChkaGLepQVDXAC071xOiK5cMF69pVA%2BbJY%3D&reserved=0)). If published, this will include your full peer review and any attached files.

If you choose “no”, your identity will remain anonymous but your review may still be made public.

**Do you want your identity to be public for this peer review?** For information about this choice, including consent withdrawal, please see our [Privacy Policy](https://nam12.safelinks.protection.outlook.com/?url=https%3A%2F%2Fwww.plos.org%2Fprivacy-policy&data=05%7C02%7Crenee.fekieta%40yale.edu%7C1d52593bc4fc4eb90d9808dcaa7ad586%7Cdd8cbebb21394df8b4114e3e87abeb5c%7C0%7C0%7C638572692034334179%7CUnknown%7CTWFpbGZsb3d8eyJWIjoiMC4wLjAwMDAiLCJQIjoiV2luMzIiLCJBTiI6Ik1haWwiLCJXVCI6Mn0%3D%7C0%7C%7C%7C&sdata=K71%2FsvC%2BUvq6yCh2aO0x9Gsg9LpSB5dQJbesGMhD5%2FI%3D&reserved=0).

Reviewer #1: **Yes:** Marcello Di Pumpo

Response: Thank you for your careful review. We are happy to include the peer review history.
